# Retrospective Diagnosis of a Novel *ACAN* Pathogenic Variant in a Family With Short Stature: A Case Report and Review of the Literature

**DOI:** 10.3389/fgene.2021.708864

**Published:** 2021-08-12

**Authors:** Valentina Mancioppi, Flavia Prodam, Simona Mellone, Roberta Ricotti, Enza Giglione, Nicolino Grasso, Denise Vurchio, Antonella Petri, Ivana Rabbone, Mara Giordano, Simonetta Bellone

**Affiliations:** ^1^Division of Pediatrics, Department of Health Sciences, University of Piemonte Orientale, Novara, Italy; ^2^Endocrinology, Department of Translational Medicine, University of Piemonte Orientale, Novara, Italy; ^3^Interdisciplinary Research Center of Autoimmune and Allergic Diseases, University of Piemonte Orientale, Novara, Italy; ^4^Laboratory of Genetics, SCDU Biochimica Clinica, Ospedale Maggiore della Carità, Novara, Italy; ^5^Department of Health Sciences, University of Piemonte Orientale, Novara, Italy

**Keywords:** short stature, *ACAN*, mutation, aggrecans, osteoarthritis dissecans

## Abstract

Short stature is a frequent disorder in the pediatric population and can be caused by multiple factors. In the last few years, the introduction of Next Generation Sequencing (NGS) in the molecular diagnostic workflow led to the discovery of mutations in novel genes causing short stature including heterozygous mutations in *ACAN* gene. It encodes for aggrecan, a primary proteoglycan component specific for the structure of the cartilage growth plate, articular and intervertebral disc. We report a novel *ACAN* heterozygous pathogenic variant in a family with idiopathic short stature, early-onset osteoarthritis and osteoarthritis dissecans (SSOAOD). We also performed a literature review summarizing the clinical characteristic of *ACAN*'s patients. The probands are two Caucasian sisters with a family history of short stature and osteoarthritis dissecans. They showed dysmorphic features such as mild midface hypoplasia, brachydactyly and broad thumbs, especially the great toes. The same phenotype was presented in the mother who had had short stature and suffered from intervertebral disc disease. DNA sequencing identified a heterozygous pathogenic variation (c.4390delG p.Val1464Ter) in the sisters, with a maternal inheritance. The nonsense mutation, located on exon 12, results in premature truncation and presumed loss of protein function. In terms of treatment, our patients underwent recombinant human growth hormone replacement therapy, associated with gonadotropin releasing hormone therapy, in order to block early growth cessation and therefore reach a better final height. Our case suggests that SSOAOD *ACAN* related should be considered in the differential diagnosis of children with autosomal dominant short stature and family history of joints disease.

## Introduction

Short stature is defined as a height of at least two standard deviations below the average observed in age and sex control population (Hauer et al., 2017). It's a frequent disorder affecting 3% of the pediatric population and one of the most common causes of pediatric endocrinologist evaluation (Stavber et al., 2020). According to its etiology, short stature can be classified into primary growth disorder, secondary growth disorder and idiopathic short stature (ISS) (Xu et al., 2018). In addition to forms determined by common variants with polygenic inheritance, recent studies have highlighted that monogenic defects could be the cause of the growth disorder observed in non-syndromic children (Grunauer and Jorge, 2018; Vasques et al., 2019). More than 700 genes are associated with growth failure, with SHOX haploinsufficiency as the most frequent form, estimated to cause 2–3% of idiopathic short stature (ISS) cases (Fukami et al., 2016).

**Figure 1:**
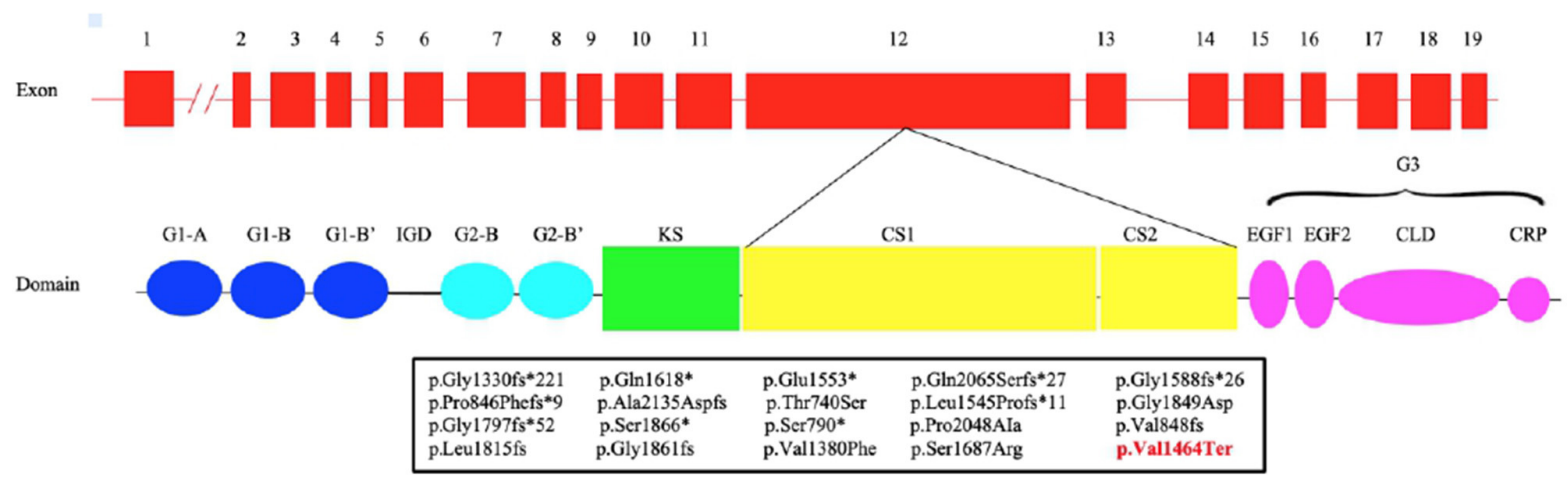
Structure of ACAN gene and aggrecan protein. Pathogenic variants in exon 12 (bottom) with respective predicted changes in the amino acid sequence are shown. The mutations identified in previous studies are shown in black, while our is in red. CLD, C-type lectin domain; CRP, complement regulatory protein-like domain; CS1, chondroitin sulfate 1; CS2, chondroitin sulfate 2; EGF1, 2, epidermal growth factor–like domain 1, 2; G1, globular domain 1; G2, globular domain 2; G3, globular domain 3; IGD, interglobular domain; KS, keratan sulfate.

The introduction of NGS in the molecular diagnostic work-flow led to the discovery of mutations in novel genes causing short stature including heterozygous mutations in *ACAN* gene (*ACAN*, OMIM:^*^155760) (Sentchordi-Montané et al., 2018). *ACAN* is located on chromosome 15q26 and consists of 19 exons, ranging in size from 77 to 4224 bp. It encodes for aggrecan, a primary proteoglycan component specific for the structure of the cartilage growth plate, articular and intervertebral disc (Uchida et al., 2020). Aggrecan is fundamental in articular cartilage, providing the hydrated gel structure necessary for the load-bearing properties of joints (Dateki, 2017) as well as chondrocyte and bone morphogenesis (Quintos et al., 2015). The core protein consists of three globular domains (G1, G2, and G3), interglobular domain (IGD), and centrally located glycosaminoglycan attachment region (GAG), consisting of chondroitin sulfate attachment domains (CS1 and CS2) and keratan sulfate attachment domain (KS) (Figure 1). G1 domain forms interactions with hyaluronan, G2 biological function is still not known whereas the G3 domain binds to different extracellular proteoglycans (i.e., tenascin and fibulin) via its C-type lectin repeat domain (CLD) (Gkourogianni et al., 2017). *ACAN* biallelic mutations are related to spondyloepimetaphyseal dysplasia, aggrecan type (SEMD, OMIM#612813) (Tompson et al., 2009), while mutations at the heterozygous state can cause spondyloepiphyseal dysplasia, Kimberley type (SEDK, OMIM#608361) (Gleghorn et al., 2005), short stature associated with or without accelerated bone age (BA), early-onset osteoarthritis (OA) and/or osteoarthritis dissecans (SSOAOD, OMIM#165800) and various idiopathic short stature phenotypes (Stattin et al., 2010; Quintos et al., 2015; Tatsi et al., 2017; Van der Steen et al., 2017).

Here we report two sisters from a family with idiopathic short stature, early-onset osteoarthritis and osteoarthritis dissecans caused by novel heterozygous *ACAN* variant.

## Case Presentation

**Figure 2:**
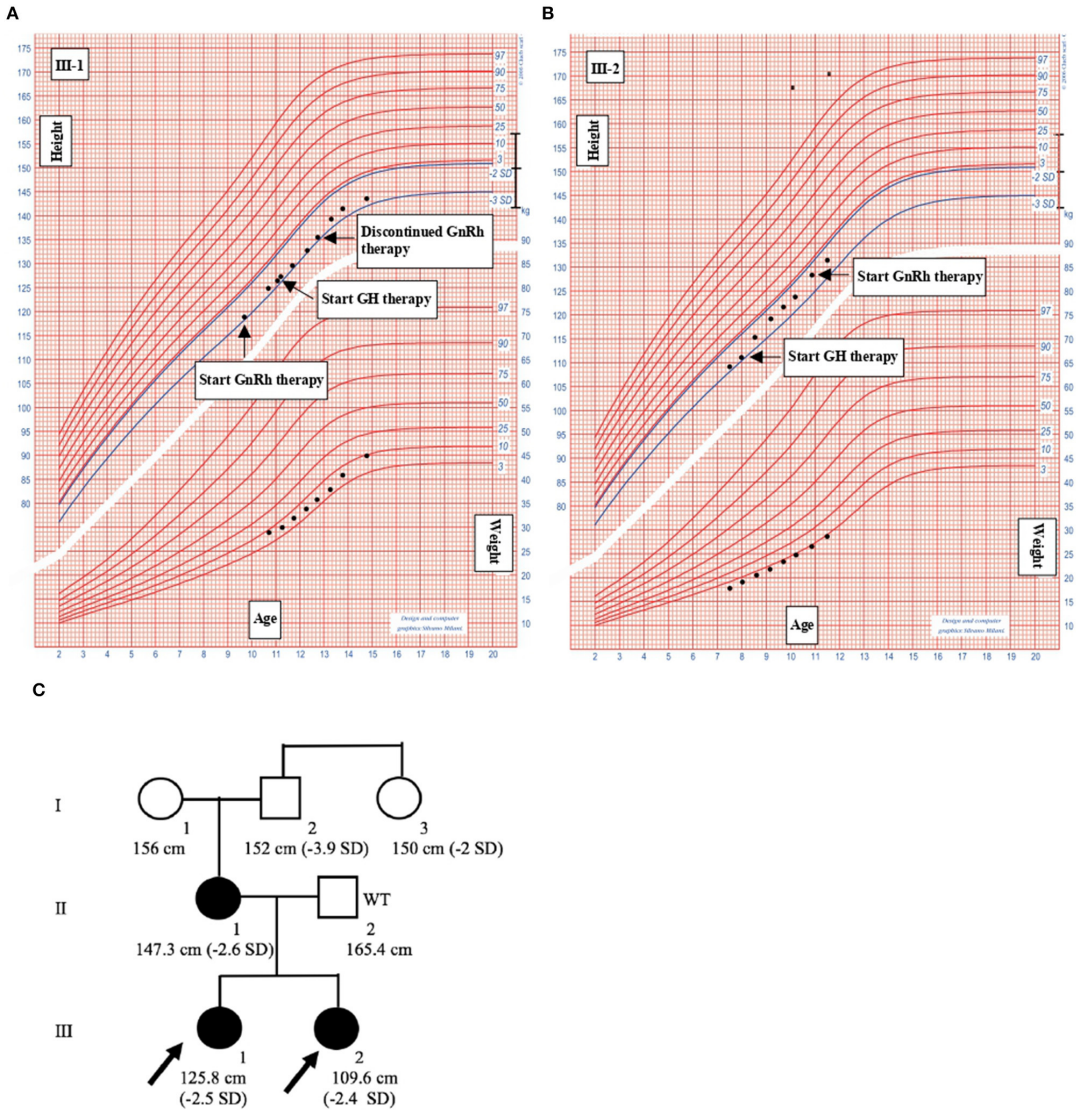
Clinical informations of the family members. **(A)** III-1. **(B)** III-2. Growth charts of the probands. Heights and weights are shown as black points. GH, Growth hormone; GnRH, gonadotropin-releasing hormone. **(C)** Pedigree of the family with familiar short stature. Shaded symbols indicate patients with stature below 2 SD; empty symbols with “WT” (Wild Type) are family members without mutations; empty symbols indicate family members who have not undergone ACAN sequencing.

The probands are two caucasian sisters (III-1 and III-2, Figures 2A,B), previously referred to another hospital for persistent short stature.

III-1 was born full-term after an uncomplicated pregnancy and delivery. At birth, her weight was 3.3 kg (−0.05 SD), length of 46 cm (−2.12 SD), head circumference of 36 cm (+1.68 SD) and she presented normal psychomotor development (Table 1). Growth rate has been below normal during most of her childhood. At our first evaluation, she was 10.8 years old, her height was 125.8 cm (−2.5 SD) with a genetic target of 150.4 cm (−2 SD). The previous year her physical examination revealed bilateral breast development (Tanner stage B2) and gonadotropin releasing hormone stimulation test was performed (peak FSH value of 18.6 mU/ml, peak PH 7.3 mU/ml). She started Gonadotropin-releasing hormone (GnRH) treatment for precocious puberty with Triptorelin 11.25 mg i.m. every 4 weeks. The father's height (II-2) was 165.4 cm (−1.84 SD) while maternal height (II-1) was 147.3 cm (−2.6 SD). Maternal family history of short stature was described: the grandfather's height (I-2) was 152 cm (−3.9 SD), the grandmother's height (I-1) was 156 cm (−1.12 SD) and the height of the grandfather's sister (I-3) was 150 cm (−2 SD) (Figure 2C). They had all the same phenotype characterized by short stature, macrocephaly, short neck, barrel chest, muscle hypertrophy and severe osteoarthritis. III-1 showed dysmorphic features such as macrocephaly, frontal bossing, low-set rotated ears, flat nasal filter, brachydactyly and broad thumbs, especially the great toes. Growth hormone (GH) stimulation test (arginine) produced a peak GH value of 25.6 ng/ml. Additional laboratory and endocrine assessment revealed normal complete blood count, chemistry panel, inflammatory markers, celiac screening, epatic-renal-thyroid function test, insulin-like growth factor-1 (IGF-1) (Table 1). The hand and wrist radiography showed no characteristic signs of skeletal dysplasia, other than shortening of the 1st distal phalanx. According to the Greulich and Pyle method, her bone age (BA) was 0.6 years advanced relative to the calendar age (CA). She started recombinant human GH (rhGH) replacement therapy at a dose of 0.25 mg/kg/week when she was 11 year old. At 12.6 years of age, the treatment with GnRH was discontinued and menarche occurred at 14.5 years of age. Moreover in the last two years, she had a history of persistent pain in both knees: x-ray and magnetic resonance imaging detected multiple osteochondral lesions consistent with osteochondritis dissecans and she underwent a surgical operation. At her latest visit (age 14.7 years) her height was 143 cm (−2.3 SD) with a growth velocity of 2 cm/year (+0.6 SD).

**Table 1:** Phenotype of our two patients with ACAN mutations.

**Patient**	**III-1**	**III-2**
**Birth characteristics**		
Weight (kg)	3.3	3.1
Length (cm)	46	46
HC (cm)	36	35.5
**First visit**		
Age (years)	10.8	7.6
Weight (kg)	28.9	18.6
Height (cm)	125.8	109.6
Target Height	150.4	150.4
Arm Span (cm)	131.5	108
Sit Height (cm)	64.5	58.9
Arm Span/height (cm)	1.04	0.99
Sit Height/height (cm)	0.51	0.54
**Endocrine evaluation**		
GH Peak at GH stimulation test (ng/ml)	25.6	27.9
IGF-1 (ng/ml)	275	132
**Skeletal system**		
Chronologic age (y)	10.6	7.6
Bone age (Greulich/Pyle)	11	7.6

*HD, head circumference; GH, growth hormone*.

The younger sister (III-2) was born full-term after an uncomplicated pregnancy with a birth weight of 3.1 kg (−0.85 SD), birth length of 46 cm (−2.34 SD), head circumference of 35.5 cm (+0.97), normal mental and motor development (Table 1). Short stature developed in early childhood, with appropriate weight for height. At our first visit, she was 7.6 years old, her height was 109.6 cm (−2.4 SD) and she was prepubertal. She had the same dysmorphic features as the older sister, bone age corresponding to chronological age, no signs of skeletal dysplasia. Growth hormone (GH) stimulation test (arginine) produced a peak GH value of 27.9 ng/ml. She had normal screening laboratory evaluation including complete blood count, inflammatory markers, celiac screening, epatic-renal-thyroid function test, insulin-like growth factor-1 (IGF-1) or other systemic explanation for her short stature (Table 1). At 8.2 years of age, she started GH therapy at a dose of 0.27 mg/kg/week. Thelarche occurred at approximately 11 years of age (height-−2.3 SD) and she was started on GnRH agonist (Triptorelin 11.25 mg i.m. every 4 weeks, still ongoing). She also suffered from persistent ankles' pain (especially the right), with the magnetic resonance imaging revealing mild osteochondritis dissecans. She didn't need to perform any surgical procedure but had to use bilateral ankle braces. At her latest visit (age 11.6 years) her height was 131.1 cm (−2.1 SD) with a growth rate of 5.3 cm/year (+1.4 SD).

After starting GH's therapy, both sisters were seen every 6 months for monitoring their growth and pubertal development but also for undergoing laboratory and endocrine assessment for glucose and lipid metabolism, epatic-renal-thyroid and corticotrope function test. All the evaluations were always normal; the dose adjustment for GH therapy was based on IGF-I levels. No side effects were registered for both GH and GnRH therapy. Clinical, anthropometric, and metabolic features during GH treatment are summarized in Table 2.

**Table 2:** Clinical, anthropometric, and metabolic features at baseline and during GH treatment.

**Clinical, anthropometric, and metabolic features**	**Baseline**		**1th year**		**2th year**		**3th year**		**4th year**
	**III-1**	**III-2**	**III-1**	**III-2**	**III-1**	**III-2**	**III-1**	**III-2**	**III-1**
Age (years)	10.8	8.2	11.7	9.1	12.7	10.1	13.8	11.6	14.7
Height (cm)	125.8	112.5	130.3	117.7	133.7	123.6	140.7	131.1	143
Weight (kg)	28.9	19.7	31.9	22.2	35.7	24.8	38.3	29.4	45.5
Tanner Stage	B1 PH1	B1 PH1	B2 PH3	B1 PH1	B2 PH3	B2 PH1	B3 PH3	B2 PH1	B4 PH4
Growth velocity (cm/year)	–	–	5.7	6.1	3.9	6.1	4.6	5.3	2.0
GH dose (mg/day)	1.0	0.7	1.0	0.8	1.3	0.8	1.3	1.0	1.6
Side effects GH therapy	N.T.	N.T.	No	no	No	no	No	no	no
IGF-1 (ng/ml)	260.2	112	419.3	300.5	589.5	294.2	678.7	304.6	480.8
HbA1c (%)	5.2	5.2	5.3	5.0	5.5	5.0	5.2	4.7	5.0
Side effects GnRH therapy	No	N.T.	No	N.T.	Therapy discontinued	N.T.	N.T.	in therapy	N.T.

*HbA1c, glycosylated hemoglobin; GH, growth hormone; IGF-1, Insulin-like growth factor-1; GnRH, gonadotropin-releasing hormone; N.T., not therapy*.

The mother had the same phenotype, characterized by short stature, macrocephaly, short neck, frontal bossing, flat nasal filter, brachydactyly and broad thumbs, especially the great toes. Despite having a stature < 2.6 SD, she was never referred to an endocrinologist or didn't undergo any endocrine assessment. From 35–40 years, she started suffering from severe intervertebral disc disease but didn't need to perform any surgical procedure.

## Methods

### Genetic Analysis

Following written informed consent, the genomic DNA of the patients was extracted from peripheral blood through the ReliaPrep Blood gDNA Miniprep System (Promega), according to the manufacturer's recommendation. The GenetiSure Dx Postnatal Array 4x180K + SNP (Agilent Technologies, USA) was used following standard protocols.

The entire coding region of the *SHOX* gene (exon 1–exon 6a/6b) and intron–exon boundaries were amplified by PCR and sequenced by Big Dye Terminator Kit (Applied Biosystems, Foster City, CA) through the automatic sequencer ABI PRISM 3100 Genetic Analyzer (Applied Biosystems). Search for deletions/duplications was performed by an MLPA assay using an MLPA Commercial Kit (SALSA MLPA Kit P018-G1 SHOX; MRC-Holland, Amsterdam, Netherlands) following the manufacturer's instructions.

A custom-designed NGS panel including 67 genes [*A2ML1, ACAN, ANKRD11, BRAF, CBL, CHD7, COL2A1, COMP, CUL7, FGD1, FGF8, FGFR1, FGFR3, FLNB GH1, GHR, GHRH, GHRHR, GHSR, GLI2, GLI3, GNAS, HDAC6, HESX1, HMGA2, HRAS, IGF1, IGF1R, IGFALS, IHH, KAT6B, KDM6A, KRAS, LHX3, LHX4, LIG4, LZTR1, MATN3, MEK1, MEK2, NF1, NPPC, NPR2, NRAS, OTX2, PCNT, PDE3A, PDE4D, POC1A, POU1F1, PROK2, PROKR2, PROP1, PTPN11, RAF1, RIT1, SHH, SHOC2, SLC26A2, SOS1, SOS2, SOX2, SOX3, SOX9, SPRED1, STAT5B, TRIM37*] involved in bone growth plate development, hypothalamic-pituitary IGF-1 axis and reported mutated in syndromic short stature was used to investigate the presence of pathogenic variants in the two sibs. An Agilent SureSelectQXT target capture method was utilized with a panel size corresponding to 1.3 Mb. The sequencing probes were designed to cover all coding exons of the 67 genes and the 20 bp flanking sequence from the intron-exon boundary. Sequencing reaction was performed using an Illumina MiSeq and MiSeq v2 300 Cycle Reagent Kits. Variant calling was performed with the SureCall v3.5 software (Agilent Technologies) and VCF files were annotated with the wANNOVAR tool. Finally post aligned, annotated, and categorized sequence data was analyzed using a personalized bioinformatics pipeline: variants were filtered by frequency, excluding those with a MAF ≥0.01 in the public databases: 1000 Genome project (https://www.internationalgenome.org/) and gnomAD (https://gnomad.broadinstitute.org/). We prioritized variants causing frameshift, stop codon, splicing or aminoacidic changes predicted as pathogenic by at least four in silico prediction software (SIFT, Polyphen-2, Mutation Taster, CADD). Classification of variants was evaluated using the consensus guidelines as set out by the American College of Medical Genetics and Genomics (ACMG) guidelines (https://www.acmg.net/ACMG). Sanger sequencing was performed to confirm the presence or absence of these mutations in all the family members.

## Results

**Figure 3:**
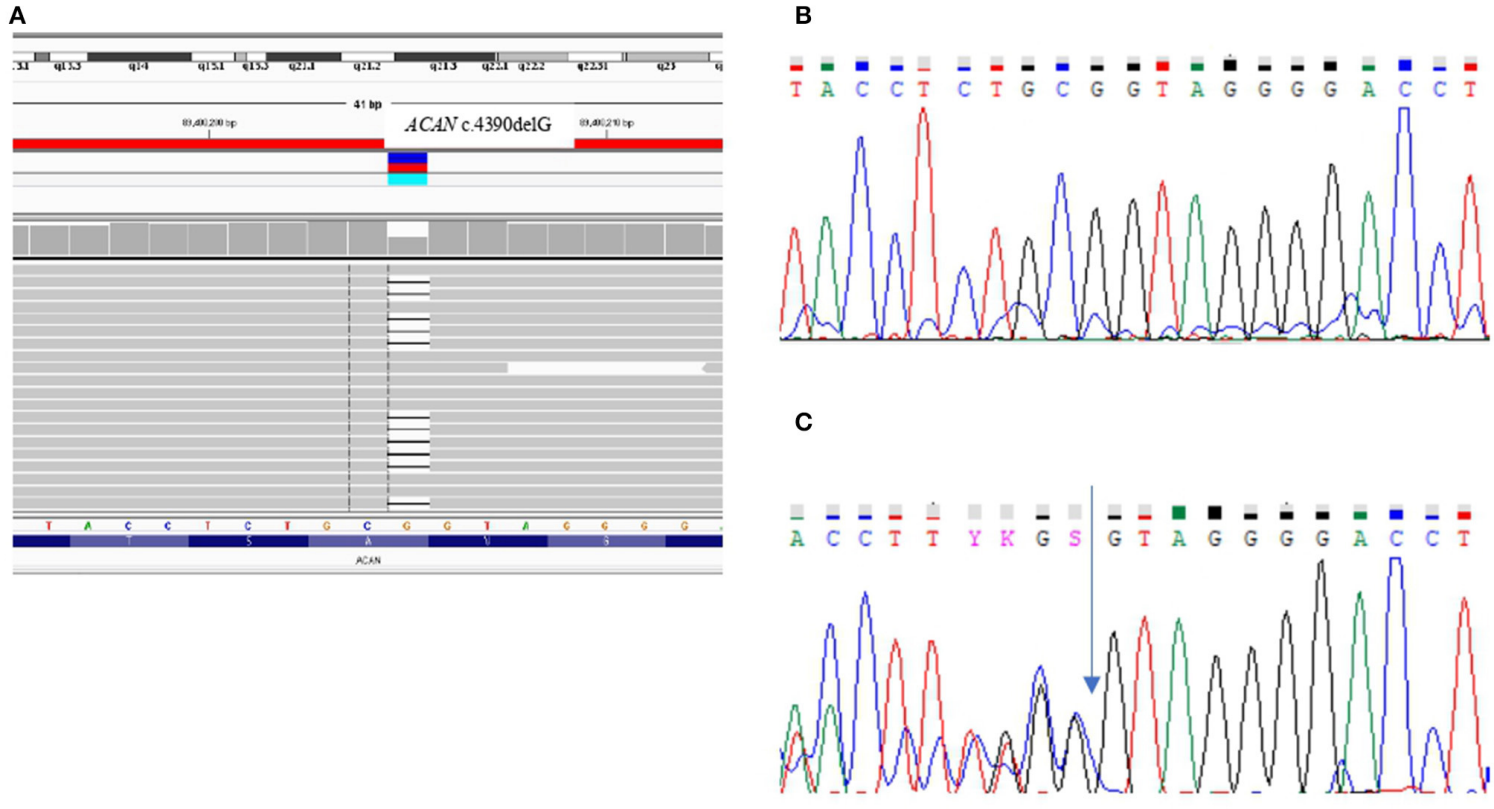
Genetic analysis. **(A)** NGS file and **(B,C)** Sanger sequencing chromatograms reporting the wild-type **(B)** and the c.4390delG variant identified in the patients **(C)** in exon 12 (NM_013227) of the ACAN gene.

In the two sisters (III-1 and III-2) the aCGH revealed a normal female Karyotype and *SHOX* molecular analysis excluded any form of SHOX-haploinsufficiency. A targeted NGS panel containing 67 genes involved in syndromic and non-syndromic short stature detected in both sibs the presence of a heterozygous pathogenic variation in exon 12, namely c.4390delG that causes the changing of the reading frame from GTA encoding Valine at position 464 to stop codon (p.Val1464Ter). The variant, validated by Sanger sequencing, was inherited from the short stature mother (Figure 3).

## Discussion and Conclusion

The etiology of short stature is complex and can be related to multiple factors such as GH deficiency, ISS, genetic inheritance, malnutrition, chronic disease (such as hypothyroidism, chronic liver and kidney diseases), constitutional delay of growth and puberty, intrauterine growth retardation, skeletal dysplasia, psychosocial stress and environmental factors (Liang et al., 2020; Wang et al., 2020). Based on new gene sequencing techniques, it is now possible to identify the cause of some short stature cases previously diagnosed as ISS.

We report a novel heterozygous mutation in *ACAN* (c.4390delG; p.Val1464Ter), located in exon 12, in a family with short stature associated with early-onset osteoarthritis and osteoarthritis dissecans (SSOAOD). Nilsson et al. (2014) reported for the first time that heterozygous *ACAN* mutations could be the cause of short stature and accelerated bone age (Nilsson et al., 2014). Since than an increasing number of *ACAN* pathogenic variants have been identified. To date, 93 mutations in this gene have been reported in HGMD (http://www.hgmd.cf.ac.uk/ac/index.php), with the majority of the variants (81/93) associated with ISS. Among them, 87.9% were inherited whereas the remaining arose *de novo*. Up to now 18 different mutations in exon 12 have been described (Figure 1; Table 3) (Gleghorn et al., 2005; Quintos et al., 2015; Gkourogianni et al., 2017; Hattori et al., 2017; Hauer et al., 2017; Tatsi et al., 2017; Van der Steen et al., 2017; Sentchordi-Montané et al., 2018; Zeng et al., 2018; Fukuhara et al., 2019; Uchida et al., 2020; Lin et al., 2021). This represents the exon with the highest number of mutations, accounting for the 19% of *ACAN* alterations (Liang et al., 2020; Lin et al., 2021). Exon 12 encodes for the chondroitin sulfate attachment domains (CS1 and CS2) and along with exon 11, for the keratan sulfate attachment domain (KS). Thanks to the sulfation of these domains (KS, CS1 and CS2) and the aggregation with hyaluronan, a fixed negative charge is generated (Dateki, 2017). This process is fundamental to cartilage because it attracts ions and water molecules, letting the cartilage bear the high mechanical load found in the skeletal joint (Gibson and Briggs, 2016). All heterozygous aggrecan mutations known so far cause short stature of similar severity, indicating that they similarly affect growth plate cartilage. Nevertheless, not all of them cause involve the articular cartilage and, therefore, determine early-onset osteoarthritis. Early-frameshift or splice-site mutations are likely to cause early truncation of the protein and may therefore functionally represent haploinsufficiency of *ACAN*. Patients with these types of mutations have short stature but no evidence of early-onset osteoarthritis, suggesting that *ACAN* haploinsufficiency primarily affects growth plate cartilage. Previously reported familial osteochondritis dissecans family and our family have missense or non-sence mutations in the C-type lectin domain and articular disease, suggesting that the presence of aggrecan protein with a dysfunctional C-type lectin domain causes a more severe phenotype affecting both growth plate and articular cartilage function (Nilsson et al., 2014). The nonsense mutation found in the present patients results in premature truncation and presumed loss of protein function. Ten frameshift variants, four missense variants and four nonsense variants (Table 3) had been previously identified in exon 12, causing a premature termination codon and the loss of the C-terminal globular domains (G3) (Figure 1; Table 3). G3 is fundamental for the proper protein function, contributing to the interaction between the aggrecan molecule and various components of the extracellular matrix (ECM) (Aspberg, 2012; Quintos et al., 2015). Nevertheless, numerous authors reiterate the importance of performing additional studies to determine whether these variants cause mRNA nonsense-mediated decay (NMD) or allow translation of a truncated aggrecan protein into the cartilage extracellular matrix (Gibson and Briggs, 2016; Stavber et al., 2020). To date, the few functional studies on the consequences of mutations in the G3 domain conducted showed the secretion of a truncated aggrecan, ruining cartilage structure and function with a dominant-negative effect (Tompson et al., 2009; Stattin et al., 2010; Gibson and Briggs, 2016).

**Table 3:** Clinical findings of patients with ACAN mutation in exon 12 in this study and the literatures.

**Subject**	**Mutation**	**Protein**	**Type**	**Sex/Age**	**Height (SDS)**	**Phenotype**	**Patterns of inheritance/affected family members (n)**	**References**
1	c.3986dupC	p.Gly1330fs*221	Frameshift	NA	NA	SEDK	NA	Gleghorn et al. (2005)
2	c.4657G>T	p.Glu1553*	Nonsense	F/NA	−3.20	ISS, midface hypoplasia, flat nasal bridge, short thumbs, OA, familiar short stature with intervertebral disc disease	Mother/(7)	Gkourogianni et al. (2017)
3	c.4762_4765del	p.Gly1588fs*26	Frameshift	M/12.3 yr	−2.70	ISS, midface hypoplasia, broad great toes, posteriorly rotated ears, familiar short stature with hip joint problems, advanteg BA (13.3 yr)	Father/(2)	Van der Steen et al. (2017)
4a	c.5391delG	p.Gly1797fs*52	Frameshift	M/5.6 yr	−2.80	ISS, midface hypoplasia, flat nasal bridge, familiar short stature with hip joint problems, advanteg BA (8 yr)	Mother/(3)	Quintos et al. (2015)
4b	c.5391delG	p.Gly1797fs*52	Frameshift	M/NA	−2.60	ISS, midface hypoplasia, flat nasal bridge, short thumbs, markedly advanteg BA, familiar short stature	Mother/(3)	Gkourogianni et al. (2017)
5	c.5597C>A	p.Ser1866*	Nonsense	M/NA	−2.00	ISS, prominent forehead, short neck, barrel-shaped chest, brachydactyly, advanteg BA	*De novo* mutation	Hauer et al. (2017)
6	c.2218A>T	p.Thr740Ser	Missense	M/3.0 yr	−3.20	ISS, frontal bossing, midface hypoplasia, high arched palate, triangular face, brachydactyly, BA equal to CA, familiar short stature	NA/(1)	Sentchordi-Montané et al. (2018)
7	c.2369C>G	p.Ser790*	Nonsense	M/14.5 yr	−2.20	ISS, frontal bossing, broad nose and philtrum, hypertelorism, brachydactyly, hyperlordosis, advanteg BA (16.5 yr), no familiar short stature,	NA/(0)	Sentchordi-Montané et al. (2018)
8	c.6142C>G	p.Pro2048Ala	Missense	F/12.5 yr	−2.20	ISS, brachydactyly, BA equal to CA, familiar short stature	NA/(1)	Sentchordi-Montané et al. (2018)
9	c.463delT	p.Leu1545Profs*11	Frameshift	F/7.8 yr	−3.60	ISS, midface hypoplasia, short left 4th toe, broad 5th toes, short 1st distal phalanx, advanteg BA (8.10 yr), familiar short stature with early-onset invertebral disc disease	Mother/(3)	Uchida et al. (2020)
10	c.2535_2536 insTTCA	p.Pro846Phefs*9	Frameshift	M/4.9 yr	−2.47	ISS, BA equal to CA, no familiar short stature	*De novo* mutation	Hattori et al. (2017)
11	c.6404delC	p.Ala2135Aspfs	Frameshift	M/6.9 yr	−3.20	Disproportionate short stature, midface hypoplasia, advanteg BA (9.9 yr), no familiar short stature	*De novo* mutation	Tatsi et al. (2017)
12	c.4852C>T	p.Gln1618*	Nonsense	M/11.7 yr	−4.00	ISS, attention deficit hyperactivity disorder, mild dysmorphic facial features as retrognathia and midface hypoplasia, BA equal to CA, familiar short stature with OA and invertebral disc disease	Father/(12)	Tatsi et al. (2017)
13a	c.6193delC	p.Gln2065Serfs*27	Frameshift	M/9.10 yr	NA	ISS, familiar short stature	Father/(3)	Zeng et al. (2018)
13b	c.6193delC	p.Gln2065Serfs*27	Frameshift	M/7 yr	NA	ISS, familiar short stature	Father/(3)	Zeng et al. (2018)
14	c.5443delC	p.Leu1815fs	Frameshift	M/4 yr	−4.38	ISS, BA equal to CA	NA	Lin et al. (2021)
15	c.5579delC	p.Gly1861fs	Frameshift	F/9.4 yr	−2.91	ISS, advanteg BA (10.7 yr),	NA	Lin et al. (2021)
16	c.4138G>T and c.5061T>A	p.Val1380Phe and p.Ser1687Arg	Missense	M/ 45 yr	−9.1	SEMD, diagnosis of osteogenesis imperfecta at 6 years of age	NA	Fukuhara et al. (2019)
17	c.2541del	p.Val848fs	Frameshift	NA	NA	ISS, OA, OCD	NA	-
18	c.5546G>A	p.Gly1849Asp	Missense	NA	NA	NA	NA	-
19a	c.4390delG	p.Val1464Ter	Nonsense	F/10.8 yr	−2.50	ISS, macrocephaly, frontal bossing, low-set rotated ears, flat nasal filter, brachydactyly and broad thumbs, OA, OCD, familiar short stature with early-onset invertebral disc disease, BA equal to CA	Mother/(3)	III-1 (current study)
19b	c.4390delG	p.Val1464Ter	Nonsense	F/7.6 yr	−2.40	ISS, macrocephaly, frontal bossing, low-set rotated ears, flat nasal filter, brachydactyly and broad thumbs, OA, OCD, familiar short stature with early-onset invertebral disc disease, BA equal to CA	Mother/(3)	III-2 (current study)

*NA, not available; Yr, year; SEDK, spondyloepiphyseal dysplasia Kimberley type; ISS, idiopathic short stature; OA, osteoarthritis; OCD, osteochondritis dissecans; SEMD, spondyloepimetaphyseal dysplasia, aggrecan type*.

The heterozygous variant detected in III-1 and III-2 was maternally inherited and their similar phenotype is consistent with that described in previous reports (Table 3), with short stature below −2.4/−2.5 SD, prognathia, flat nasal bridge, midfacial hypoplasia, posteriorly rotated ears, broad forehead, relative macrocephaly, brachydactyly, broad great toes, short thumbs, lordosis, genu valgum, joint problems (Dateki, 2017; Dateki et al., 2017; Hu et al., 2017; Van der Steen et al., 2017; Kim et al., 2020; Nilsson, 2020). The same characteristics can be detected, also, among patients carrying *ACAN* mutations located on other domains, underlying the absence of a genotype-phenotype correlation. The three most frequent features described among all *ACAN*'s patients were short neck, mild midface hypoplasia (frontal bossing, flat nasal bridge, and long philtrum), and brachydactyly, secondly osteoarthritis, spine malformation, short limbs, thoracic deformity (Liang et al., 2020). Both the sibs developed early-onset osteoarthritis (II-1 in the knee, while the III-2 in the ankle) and, subsequently, osteochondritis dissecans. While these clinical presentations are relatively common in patients carrying *ACAN* mutations, in the other cases with truncating mutations in the G3 domain there were only two cases of early-onset osteoarthritis (Gleghorn et al., 2005; Gkourogianni et al., 2017) but no other osteochondritis dissecans. The two sibs presented joint problems at a young age (between 10–12 years), in contrast to previous reports where the common start was late adolescence or even later (Gkourogianni et al., 2017). Their affected mother (II-1) suffered from severe intervertebral disc disease, starting at 35–40 years, indicating wide phenotype variability within this family.

In terms of treatment, although the two sisters didn't develop precocious puberty, they underwent gonadotropin releasing hormone (GnRH) therapy, to block early growth cessation and therefore reached a better final height. Several studies evaluated the effectiveness of combined GH and GnRH analog treatment for achieving a proper final height in patients with *ACAN* mutations (Dateki, 2017; Van der Steen et al., 2017; Zeng et al., 2018; Stavber et al., 2020). Gkourogianni et al. (2017) found that the mean height SD for *ACAN* adult patients previously treated with GH was −2.5, while that of untreated adult individuals was −3.0 (Gkourogianni et al., 2017). Furthermore, Van der Steen et al. (2017) reported that GH-treated adult individuals, who received in addition GnRH analog treatment for two years from puberty (mean age of GnRH treatment's start between 10–12 years, with normal timing of puberty), gain 5–8 cm at their adult height compared to their family members, with the same sex and *ACAN* mutation (Van der Steen et al., 2017). Stavber et al. (2020) described the cases of three children who, in addition to GH treatment, received treatment with GnRHa during puberty after 10 years of age. During the combined therapy there was a significant improvement in SD height and growth velocity increased significantly. Nevertheless, the author underlines that it's not possible to establish the efficacy of the therapy since none of the patients have already achieved the final height at the time of publication (Stavber et al., 2020). Therefore, it is still controversial if *ACAN* patients need to undergo GH therapy with or without GnRHa to achieve a proper final height and further studies are needed (Dateki, 2017).

In conclusion we identified a novel heterozygous *ACAN* mutation causing SSOAOD in the family. SSOAOD *ACAN* related should be considered in the differential diagnosis of children with autosomal dominant short stature and family history of joints disease, even in patients in which BA is equal to CA. Diagnosis of the condition is difficult, and genetic testing may help. As for treatment, growth therapies could be beneficial for improving affected individuals' adult height.

## Data Availability Statement

The datasets for this article are not publicly available due to concerns regarding participant/patient anonymity. Requests to access the datasets should be directed to the corresponding author.

## Author Contributions

VM collected the anamnestic as well as the biochemical data for the patients. VM performed the literature search, reviewed and extracted data from the papers. MG and SM performed the genetic analysis. VM performed the figures and table designing and the manuscript writing with the assistance of MG, SB, and FP. All authors discussed the results and contributed to the final manuscript.

## Conflict of Interest

The authors declare that the research was conducted in the absence of any commercial or financial relationships that could be construed as a potential conflict of interest.

## Publisher's Note

All claims expressed in this article are solely those of the authors and do not necessarily represent those of their affiliated organizations, or those of the publisher, the editors and the reviewers. Any product that may be evaluated in this article, or claim that may be made by its manufacturer, is not guaranteed or endorsed by the publisher.

## References

[B1] Aspberg A. (2012). The different roles of aggrecan interaction domains. J. Histochem. Cytochem. 60, 987–996. 10.1369/0022155412464376 23019016 PMC3527881

[B2] Dateki S. (2017). ACAN mutations as a cause of familial short stature. Clin. Pediatr. Endocrinol. 26, 119–125. 10.1297/cpe.26.119 28804204 PMC5537209

[B3] Dateki S. Nakatomi A. Watanabe S. Shimizu H. Inoue Y. Baba H.. (2017). Identification of a novel heterozygous mutation of the Aggrecan gene in a family with idiopathic short stature and multiple intervertebral disc herniation. J. Hum. Genet. 62, 717–721. 10.1038/jhg.2017.33 28331218

[B4] Fukami M. Seki A. Ogata T. (2016). SHOX Haploinsufficiency as a cause of syndromic and nonsyndromic short stature. Mol. Syndromol. 7, 3–11. 10.1159/000444596 27194967 PMC4862394

[B5] Fukuhara Y. Cho S. Y. Miyazaki O. Hattori A. Seo J. H. Mashima R.. (2019). The second report on spondyloepimetaphyseal dysplasia, aggrecan type: a milder phenotype than originally reported. Clin. Dysmorphol. 28, 26–29. 10.1097/MCD.0000000000000241 30124491 PMC6276860

[B6] Gibson B. G. Briggs M. D. (2016). The aggrecanopathies; an evolving phenotypic spectrum of human genetic skeletal diseases. Orphanet J. Rare Dis. 11:86. 10.1186/s13023-016-0459-2 27353333 PMC4924316

[B7] Gkourogianni A. Andrew M. Tyzinski L. Crocker M. Douglas J. Dunbar N.. (2017). Clinical characterization of patients with autosomal dominant short stature due to aggrecan mutations. J. Clin. Endocrinol. Metab. 102, 460–469. 10.1210/jc.2016-3313 27870580 PMC5413162

[B8] Gleghorn L. Ramesar R. Beighton P. Wallis G. (2005). A mutation in the variable repeat region of the aggrecan gene (AGC1) causes a form of spondyloepiphyseal dysplasia associated with severe, premature osteoarthritis. Am. J. Hum. Genet. 77, 484–490. 10.1086/444401 16080123 PMC1226213

[B9] Grunauer M. Jorge A. A. L. (2018). Genetic short stature. Growth Horm. IGF Res. 38, 29–33. 10.1016/j.ghir.2017.12.003 29249624

[B10] Hattori A. Katoh-Fukui Y. Nakamura A. Matsubara K. Kamimaki T. Tanaka H.. (2017). Next generation sequencing-based mutation screening of 86 patients with idiopathic short stature. Endocr. J. 64(10), 947–954. 10.1507/endocrj.EJ17-0150 28768959

[B11] Hauer N. N. Sticht H. Boppudi S. Büttner C. Kraus C. Trautmann U.. (2017). Genetic screening confirms heterozygous mutations in ACAN as a major cause of idiopathic short stature. Sci Rep. 22:12225. 10.1038/s41598-017-12465-6 28939912 PMC5610314

[B12] Hu X. Gui B. Su J. Li H. Li N. Yu T.. (2017). Novel pathogenic ACAN variants in non-syndromic short stature patients. Clin. Chim. Acta 469, 126–129. 10.1016/j.cca.2017.04.004 28396070

[B13] Kim Y. T. Jang K. M. Keum C. W. Oh S. H. Chung W. Y. (2020). Identification of a heterozygous ACAN mutation in a 15-year-old boy with short stature who presented with advanced bone age: a case report and review of the literature. Ann. Pediatr. Endocrinol. Metabol. 25, 272–276. 10.6065/apem.1938198.099 32871652 PMC7788345

[B14] Liang H. Miao H. Pan H. Yang H. Gong F. Duan L.. (2020). Growth-promoting therapies may be useful in short stature patients with nonspecific skeletal abnormalities caused by Acan heterozygous mutations: six Chinese cases and literature review. Endocr. Pract. 26, 1255–1268. 10.4158/EP-2019-0518 33471655

[B15] Lin L. Li M. Luo J. Li P. Zhou S. Yang Y.. (2021). A high proportion of novel ACAN mutations and their prevalence in a large cohort of Chinese short stature children. J. Clin. Endocrinol. Metab. 106(7):e2711–e2719. 10.1210/clinem/dgab088 33606014 PMC8208663

[B16] Nilsson O. (2020). Aggrecanopathies highlight the need for genetic evaluation of ISS children. Eur J. Endocrinol. 183:C9–C10. 10.1530/EJE-20-0420 32413843

[B17] Nilsson O. Guo M. H. Dunbar N. Popovic J. Flynn D. Jacobsen C.. (2014). Short stature, accelerated bone maturation, and early growth cessation due to heterozygous aggrecan mutations. J. Clin. Endocrinol. Metab. 99, E1510–E1518. 10.1210/jc.2014-1332 24762113 PMC4121031

[B18] Quintos J. B. Guo M. H. Dauber A. (2015). Idiopathic short stature due to novel heterozygous mutation of the aggrecan gene. J. Pediatr. Endocrinol. Metab. 28, 927–932. 10.1515/jpem-2014-0450 25741789 PMC4501863

[B19] Sentchordi-Montané L. Aza-Carmona M. Benito-Sanz S. Barreda-Bonis A. C. Sánchez-Garre C. Prieto-Matos P.. (2018). Heterozygous aggrecan variants are associated with short stature and brachydactyly: description of 16 probands and a review of the literature. Clin. Endocrinol. (Oxf). 88, 820–829. 10.1111/cen.13581 29464738

[B20] Stattin E. L. Wiklund F. Lindblom K. Onnerfjord P. Jonsson B. A. Tegner Y.. (2010). A missense mutation in the aggrecan C-type lectin domain disrupts extracellular matrix interactions and causes dominant familial osteochondritis dissecans. Am. J. Hum. Genet. 86, 126–137. 10.1016/j.ajhg.2009.12.018 20137779 PMC2820178

[B21] Stavber L. Hovnik T. Kotnik P. Lovrečić L. Kovač J. Tesovnik T.. (2020). High frequency of pathogenic ACAN variants including an intragenic deletion in selected individuals with short stature. Eur. J. Endocrinol. 182, 243–253. 10.1530/EJE-19-0771 31841439 PMC7087498

[B22] Tatsi C. Gkourogianni A. Mohnike K. DeArment D. Witchel S. Andrade A. C.. (2017). Aggrecan mutations in nonfamilial short stature and short stature without accelerated skeletal maturation. J. Endocr. Soc. 1, 1006–1011. 10.1210/js.2017-00229 29264551 PMC5686699

[B23] Tompson S. W. Merriman B. Funari V. A. Fresquet M. Lachman R. S. Rimoin D. L.. (2009). A recessive skeletal dysplasia, SEMD aggrecan type, results from a missense mutation affecting the C-type lectin domain of aggrecan. Am. J. Hum. Genet. 84, 72–79. 10.1016/j.ajhg.2008.12.001 19110214 PMC2668039

[B24] Uchida N. Shibata H. Nishimura G. Hasegawa T. (2020). A novel mutation in the ACAN gene in a family with autosomal dominant short stature and intervertebral disc disease. Hum. Genome Var. 7:44. 10.1038/s41439-020-00132-8 33298914 PMC7712780

[B25] Van der Steen M. Pfundt R. Maas S. J. W. H. Bakker-van Waarde W. M. Odink R. J. Hokken-Koelega A. C. S. (2017). ACAN gene mutations in short children born SGA and response to growth hormone treatment. J. Clin. Endocrinol. Metab. 102, 1458–1467. 10.1210/jc.2016-2941 27710243

[B26] Vasques G. A. Andrade N. L. M. Jorge A. A. L. (2019). Genetic causes of isolated short stature. Arch. Endocrinol. Metab. 63, 70–78. 10.20945/2359-3997000000105 30864634 PMC10118839

[B27] Wang Y. Ge J. Ma J. Qiao L. Li T. (2020). Short stature with precocious puberty caused by aggrecan gene mutation: a case report. Medicine (Baltimore) 99:e21635. 10.1097/MD.0000000000021635 32846772 PMC7447401

[B28] Xu D. Sun C. Zhou Z. Wu B. Yang L. Chang Z.. (2018). Novel aggrecan variant, p. Gln2364Pro, causes severe familial nonsyndromic adult short stature and poor growth hormone response in Chinese children. BMC Med. Genet. 19:79. 10.1186/s12881-018-0591-z 29769040 PMC5956957

[B29] Zeng T. Liao L. Y. Li N. Wang J. Peng J. Guo Y.. (2018). Familial short stature caused by ACAN gene mutation: a familial case report [in Chinese]. J. Clin. Pediatr. 36, 463–466. 10.3969/j.issn.1000-3606.2018.06.015

